# The First Described Nymphs and Detailed Imagoes of the Species *Thalerosphyrus cingulatus* Navás Revealing a New Mayfly Genus from Eastern China (Ephemeroptera: Heptageniidae, Ecdyonurinae) †

**DOI:** 10.3390/insects12111020

**Published:** 2021-11-12

**Authors:** Zhiming Lei, Dewen Gong, Wei Zhang, Changfa Zhou

**Affiliations:** The Key Laboratory of Jiangsu Biodiversity and Biotechnology, College of Life Sciences, Nanjing Normal University, Nanjing 210023, China; zrqzop@gmail.com (Z.L.); nnudwyane@163.com (D.G.); 171201004@njnu.edu.cn (W.Z.)

**Keywords:** heptageniid mayfly, *Regulaneuria* gen. nov., morphology, classification, insect

## Abstract

**Simple Summary:**

The reduction or loss of crossveins on colorful wings can be found in several lineages of mayflies, especially those with small to tiny bodies. However, in the family Heptageniidae, whose nymphs have very flat bodies and usually crawl on the surface of rocks to pebbles, this situation is very rare. Surprisingly, about 90 years ago, a Spanish entomologist Longinos Navás briefly described and illustrated a Chinese mayfly, *Thalerosphyrus cingulatus*, with few crossvein rows which is further heavily pigmented with yellowish to brown stains. Unfortunately, this interesting species and its outstanding morphology have never been re-checked or confirmed until now. In 2021, tens of specimens of this mayfly were re-found and its mating flight behavior was recorded. Therefore, the original characters of this species are re-described and photographed in detail. The results show both its imagoes and nymphs have a long series of combined characteristics of several related genera, and it represents a new clade and owns a new phylogenetic position of the subfamily Ecdyonurinae. To reflect its uniqueness, a new genus, *Regulaneuria* Zhou, gen. nov., is established herein. The similar venations are found in several families of the order Ephemeroptera. This means the reduction of crossveins can happen repeatedly and convergently in mayflies but our finding here is the extreme one of the family Heptageniidae.

**Abstract:**

The nymph and detailed imaginal morphology of the Chinese mayfly *Thalerosphyrus cingulatus* have not been reported since it was named by Navás in 1933. Here based on newly collected nymphal and associated imaginal materials of this species from eastern China, we find both nymphs and adults have several extraordinary characters. In adults, forewings have less crossveins in costal and subcostal sections, others crossveins arranged into five regular rows; tibiae and tarsi of midlegs and hindlegs subequal in length; male compound eyes widely separated and penes simple and fused. The nymphs have greatly extended lateral pronotum, round supracoxal spurs and extended dorsal lamellae of gills, especially those of the gills VII; maxillae have two independent distal dentisetae. These combined characters represent a new generic taxon. Therefore, *Regulaneuria* Zhou, gen. nov. is established here to include the species *Regulaneuria cingulata* (Navás, 1933) comb. nov. The forewing venation of the species *R*. *cingulata* is unique in the family Heptageniidae but similar to some counterparts of Leptophlebiidae and Baetidae.

## 1. Introduction

Navás named the species *Thalerosphyrus cingulatus* from Chusan (now spelling as Zhoushan), China [1]. The original description and drawing on forewing show this species has greatly reduced crossveins and relatively long hindtibiae. However, because the types in Heude Museum is lost [2], this species has never been re-described although it was cited by Wu, Gui, Zhou and Zhou et al. [3,4,5,6], and was transferred to the genus *Compsoneuria* Eaton, 1881 by Braasch and Soldán and also mentioned by Webb et al. [7,8,9], but this arragement was doubted by Sartori [8]. No further information has been added until now.

In June and July 2021, tens of nymphs and adults of this species were found and collected in Ningbo and Wenzhou cities, Zhejiang Province, Eastern China, both places near the location where the types were caught (Chusan). After careful examination and comparison, we believe it represents a new genus of the family Heptageniidae. Thus, all stages of it are described and photographed in detail herein. Additionally, brief ecological notes of this species are also provided. Similar to the genus *Compsoneuria*, the imagoes of this species have reduced crossveins on wings but it goes further. The crossveins of forewings are aligned into 4–5 rows. On the other side, the nymphal characters of this species show the new genus has a combined morphology of several related genera of Heptageniidae.

## 2. Materials and Methods

The nymphs were collected by hand net and adults were caught by air net during daytime. Some adults were reared indoor from mature nymphs or attracted by light at night. All materials were stored in ethanol (more than 80%).

All specimens were photographed with a digital camera (Single Lens Reflex, Guangzhou, China) and examined under a stereomicroscope. Some small structures, such as mouthparts, claws and gills were observed and photographed with a microscope camera.

Eggs were obtained from female imagoes. Eggs and maxillae were scanned by SEM (Scanning Electron Microscope, Model JSM-5610LV, Tokyo, Japan). All SEM samples were prepared with a standard protocol: fixed in 4% glutaraldehyde for 5–8 h, rinsed with physiological saline 2–3 times (10–15 min each), dehydrated in concentration gradient acetone (30%, 50%, 70%, 80%, 90%, 100%, 10 to 15 min each), and coated with gold film in a vacuum.

All specimens used in this study are deposited in the Mayfly collection, College of Life Sciences, Nanjing Normal University (NNU).

Abbreviations used in this study are as follows: C (costal); KCT (knob-terminated coiled threads); MA (Medius anterior); MP (Medius posterior); Rs (Radial sector); R_1_ (Radius); Sc (Subcosta).

## 3. Results

### 3.1. Regulaneuria gen. nov. Zhou

Type species and species included: *Regulaneuria cingulata* (Navás, 1933), new combination.

### 3.2. Genus Definition

**Figure 1:**
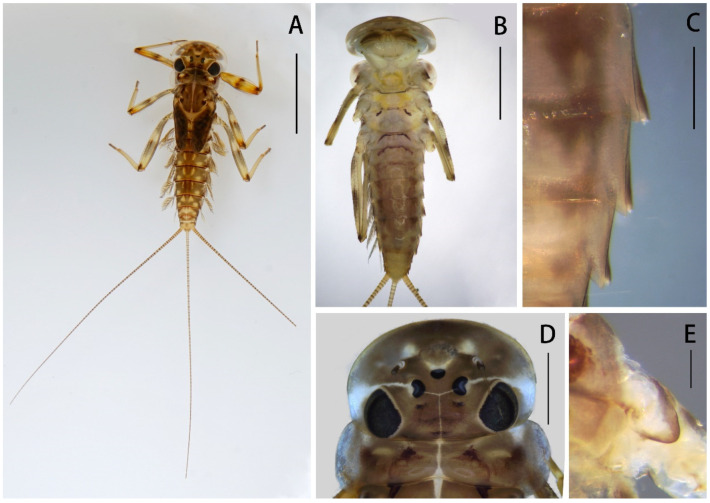
Nymph of *Regulaneuria cingulata*: (**A**) Nymphal habitus; (**B**) Ventral surface of nymph; (**C**) Posterolateral projections of terga V–VII (ventral view); (**D**) Head capsule and pronotum; (**E**) Supracoxal spur (midleg). Scale bars: (**A**) = 5.0 mm; (**B**) = 2.0 mm; (**C**) = 0.5 mm; (**D**) = 1.0 mm; (**E**) = 0.1 mm.

**Figure 2:**
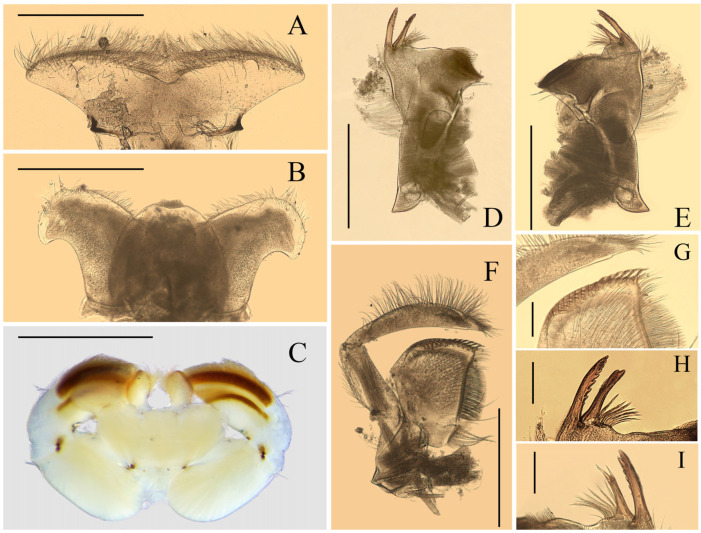
Mouthparts of *Regulaneuria cingulata*: (**A**) Labrum (ventral view); (**B**) Hypopharynx (ventral view); (**C**) Labium (ventral view); (**D**) Left mandible (ventral view); (**E**) Right mandible (ventral view); (**F**) Maxillae; (**G**) Apex of maxillae; (**H**) Incisors of left mandible; (**I**) Incisors of right mandible. Scale bars: (**A**,**B**,**D**–**F**) = 0.5 mm; (**C**) = 1.0 mm; (**G**) = 0.2 mm; (**H**,**I**) = 0.1 mm.

**Figure 3:**
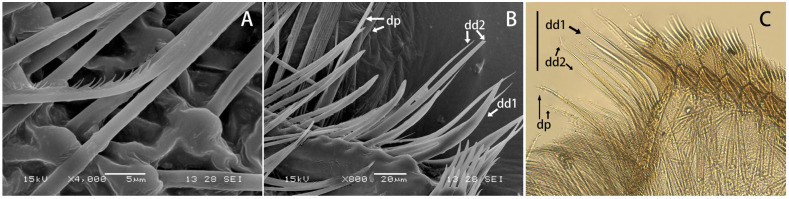
Maxillae of *Regulaneuria cingulata* (**A**) SEM view of fimbriate scattered setae on the ventral face of the galea; (**B**) SEM view of the distal dentisetae and proximal dentiseta; (**C**) Same view in optical microscope. Scale bars: (**C**) = 0.5 mm.

**Figure 4:**
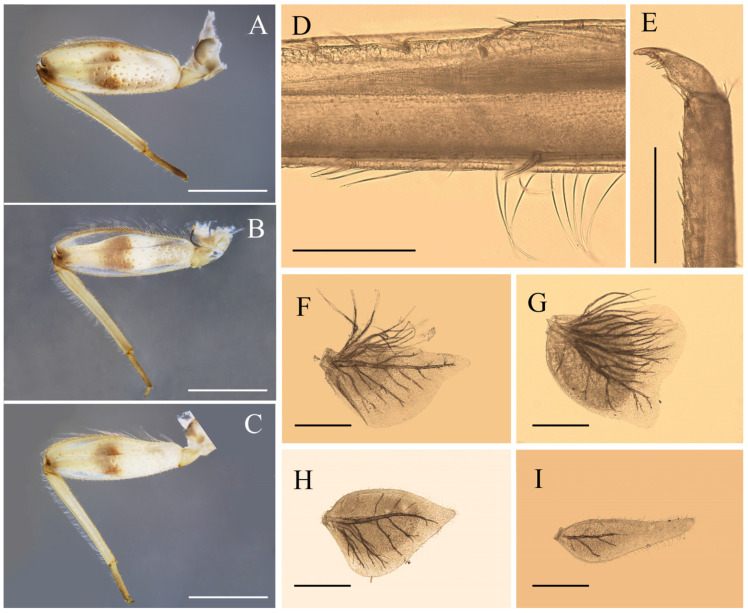
Nymphal structures of *Regulaneuria cingulata*: (**A**) Foreleg; (**B**) Midleg; (**C**) Hindleg; (**D**) Hindleg tibia; (**E**) Foreleg claw; (**F**) Gill I; (**G**) Gill II; (**H**) Gill VI; (**I**) Gill VII. Scale bars: (**A**–**C**) = 1.0 mm; (**D**) = 0.2 mm; (**E**) = 0.5 mm; (**F**–**I**) = 0.2 mm.

Nymph: pronotum expanded laterally, wider than head (Figure 1D); labrum ca. half of head width, with round lateral apex (Figure 2A); superlinguae of hypopharynx extended posterolaterally into bent blunt apex (Figure 2B); maxillae with two distal dentisetae (second one bifurcated) and one proximal dentiseta (with two branches, second one fringed) (Figure 3B,C); supracoxal spurs well-developed, round (Figure 1E); lamellae of gills I–VI extended into projections (Figure 4F–H), gills VII with lamellae only, near leaf-like in shape (Figure 4I); caudal filaments with spines on articulations.

**Figure 5:**
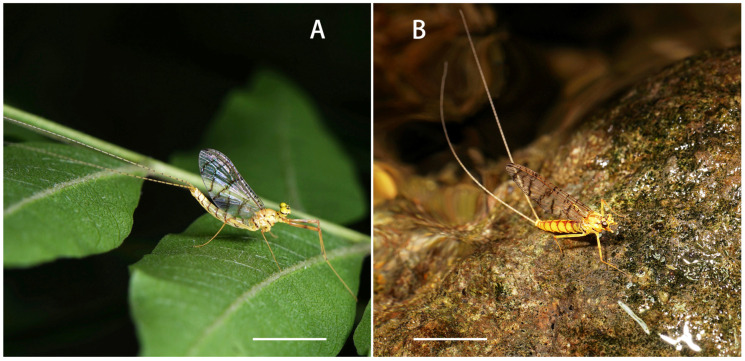
Adults of *Regulaneuria cingulata*: (**A**) Male imago habitus; (**B**) Female imago habitus. Scale bars: (**A**,**B**) = 5.0 mm.

**Figure 6:**
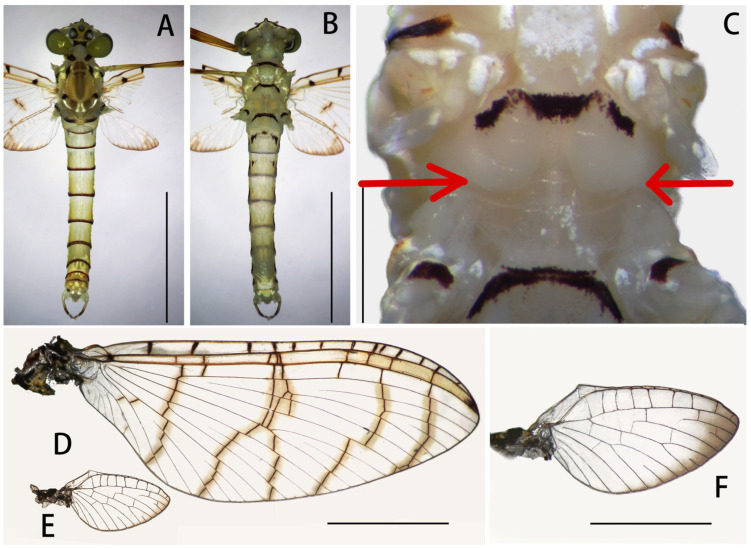
Male structures of *Regulaneuria cingulata*: (**A**) Male imago (dorsal view); (**B**) Male imago (ventral view); (**C**) Medial depression of furcasternum (shown in red arrow); (**D**) Forewing; (**E**) Hindwing; (**F**) Hindwing enlarged. Scale bars: (**A**,**B**) = 3.0 mm; (**C**) = 1.0 mm; (**D**,**E**) = 2.0 mm; (**F**) = 1.0 mm.

**Figure 7:**
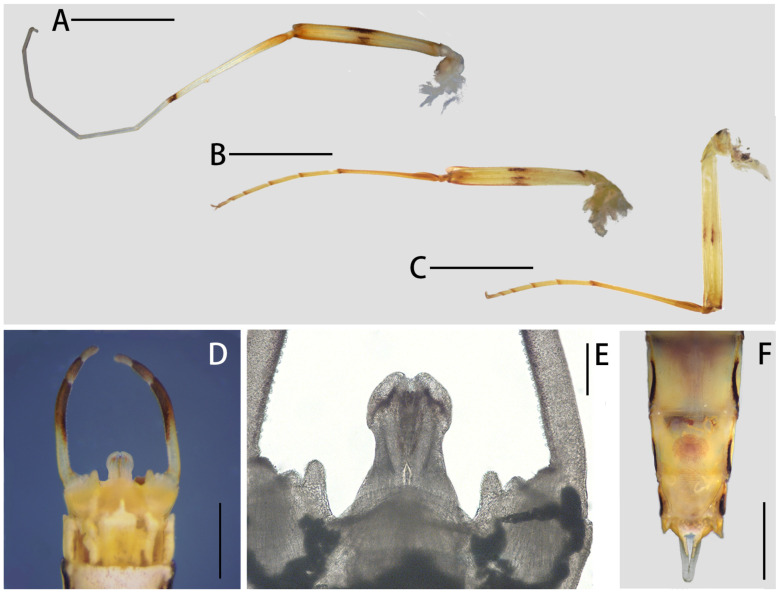
Adult structures of *Regulaneuria cingulata*: (**A**) Foreleg of male; (**B**) Midleg of male; (**C**) Hindleg of male; (**D**) Genitalia; (**E**) Penes enlarged (ventral view); (**F**) Subgenital and subanal plates of female (ventral view). Scale bars: (**A**–**C**) = 2.0 mm; (**D**) = 0.5 mm; (**E**) = 0.2 mm; (**F**) = 2.0 mm.

**Figure 8:**
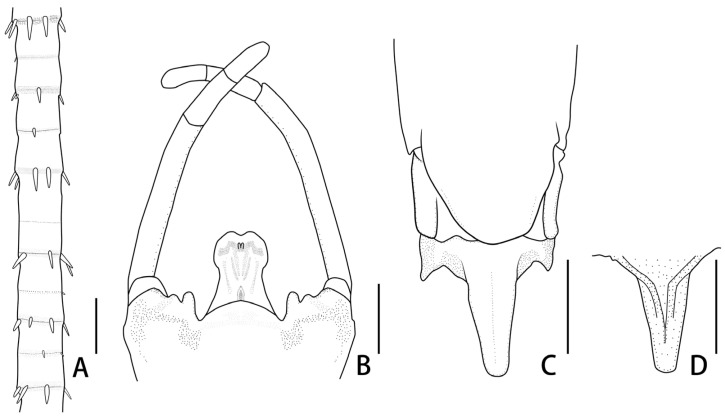
Nymphal and imaginal structures of *Regulaneuria cingulata*: (**A**) Whorls of spines of caudal filaments; (**B**) Genitalia (ventral view); (**C**) Subgenital and subanal plates of female imago (ventral view); (**D**) Subanal plate of female imago (dorsal view). Scale bars: (**A**,**B**) = 0.2 mm; (**C**,**D**) = 0.5 mm.

Imago: distance between two compound eyes of males 3× diameter of middle ocelli (Figure 5A and Figure 6A); numbers of crossveins on forewings reduced (less than 15 in C and Sc sections), all crossveins obviously pigmented in yellowish brown; except those in C and Sc fields and wingbase, all other crossveins of forewings arranged into five rows (Figure 6D); length of hindwings 1.5× of width, with brownish pigmented margins (Figure 6E); first tarsal segment of male forelegs 0.7× second one (Figure 7A); tarsi of mid- and hindlegs subequal to tibiae in length (Figure 7B,C); styliger plate convex, posterior margin with two remarkable lateral projections, penes entirely fused, inner median titillators present (Figure 7D,E and Figure 8B). Female subgenital and subanal plates greatly extended posteriorly (Figure 7F and Figure 8C,D).

**Figure 9:**
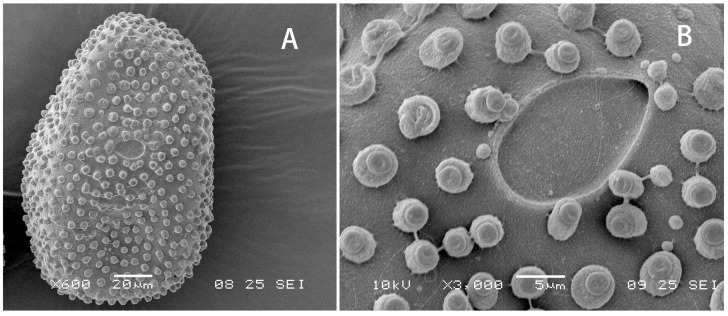
Egg of *Regulaneuria cingulata*: (**A**) Whole picture; (**B**) Micropyle and small KCTs enlarged.

Egg: Ovoid, chorion decorated randomly with irregularly shaped small KCTs on whole surface (Figure 9A). Micropyle oval and located equatorially (Figure 9B).

Etymology: The genus name *Regulaneuria* is feminine, from Latin word “*Regula*-” (regular) and “-*neuria*” (vein). It indicates the imagoes of this species having few regularly aligned crossveins on forewings.

Diagnosis: upon the anteriorly divergent medial depression of mesothoracic furcasternum in imagoes and scattered setae on the ventral surface of the maxillae, obviously this new genus is a member of the subfamily Ecdyonurinae according to the definition of Kluge [10] and the key of Webb and McCafferty [11].

Regarding imaginal reduced crossveins of C and Sc sections on forewings and nymphal two independent distal dentisetae and similar proximal dentiseta (with a fringed branch), our new genus is related to the three genera (*Compsoneuria* Eaton, 1881, *Compsoneuriella* Ulmer, 1939 and *Notonurus* Crass, 1947) of Compsoneuriini *sensu* Wang and McCafferty (2004) and Sartori (2014) [8,12,13,14,15]. However, in contrast to the two Oriental genera *Compsoneuria* and *Compsoneuriella*, our new genus can be differentiated from the former one by longer first segment of foretarsi (ca. 0.7× of second one), colorful wings and regularly aligned crossveins; in nymph, by narrower labrum, acute tips of hypopharynx superlinguae, extended gill lamellae and pronotum, forked second distal dentisetae of maxillae. In contrast with the genus *Compsoneuriella*, our new genus has less crossveins of forewings and fused penes. The nymphal characters mentioned above are also good features to separate these two genera and their supracoxal spurs are also different (acute in *Compsoneuriella* but blunt in *Regulaneuria*). The Afrotropical genus *Notonurus* is different to our new genus *Regulaneuria* in multi-branched distal dentisetae and simple scattered setae on the maxillae in nymphal stage. In imaginal stage, their penes are divergent apically and crossveins are randomly distributed [8].

Generally, both imagoes and nymphs of our new genus *Regulaneuria* gen. nov. are similar to those of *Asionurus* Braasch and Soldán, 1986a in some way, such as imaginal genitalia (almost totally fused penes with tiny titillators) and nymphal elongated gills VII, bent tips of hypopharynx superlinguae, expanded laterally pronotum [7]. However, the imagoes of the latter genus have more randomly arranged crossveins on forewings and shorter tarsi of mid-and hindlegs than the former one. In the nymphal stages, two genera can be separated by the tips of hypopharynx superlinguae (acute in *Asionurus* but blunt in *Regulaneuria* gen. nov.), labrum (greatly extended in *Asionurus* while only slightly extended in *Regulaneuria*) and gills I–V (with smooth outlines in *Asionurus* but with waved posterior margins in *Regulaneuria*).

*Regulaneuria* gen. nov. has several common characters of different related genera: the widely separated compound eyes (such as *Leucrocuta* Flowers, 1980 and *Stenacron* Jensen, 1972 [16,17], for example, *Leucrocuta hebe* (McDunnough, 1924) and *Stenacron carolina* (Banks, 1914) [18,19]); remarkably reduced crossveins of forewings (as in some species in the genera *Compsoneuria* and *Compsoneuriella*, e.g., *Compsoneuria spectabilis* Eaton, 1881 and *Compsoneuriella braaschi* Boonsoong and Sartori, 2015) [12,20]; hindwings with pigmented margins (similar to *Atopopus* Eaton, 1881 [12], such as *Atopopus edmundsi* Wang and McCafferty, 1995) [21]; subequal tarsi and tibiae of hindlegs (similar to Compsoneuriini, *Thalerosphyrus* Eaton, 1881 and some species of *Epeorus* Eaton, 1881, such as *E*. *melli* Ulmer, 1925) [12]. Male genitalia, female subanal plate, nymphal gills VII and hypopharynx of this new genus is close to *Asionurus* Braasch and Soldán, 1986 (e.g., *A. primus* Braasch and Soldán, 1986b) [22]. The morphology of proximal dentiseta of our new genus such as those of the genera *Compsoneuria* and *Compsoneuriella* [8].

In recent years, more heptageniid eggs are scanned [23,24,25]. Although without any recent comprehensive review on heptageniid eggs, we can compare them preliminarily among related genera. Due to being without any polar cap and similar knob-terminated coiled threads in size and position, the eggs of our new genus *Regulaneuria* is alike those of *Ecdyonurus* in some level [25]. However, the knob-terminated coiled threads of the former are less and bigger than the latter. The threads on eggs of the *Asionurus*, *Notacanthurus* and *Thalerosphyrus* are further smaller than those of *Ecdyonurus* [26]. In contrast, the eggs of *Compsoneuria* and *Compsoneuriella* have much larger and denser coiled threads on surface than those of *Regulaneuria*. The eggs of *Afronurus* have large coiled threads on equatorial portion.

Among above combinations, three characters are regarded as the key identification ones of our new genus: greatly reduced, regularly aligned and pigmented crossveins of forewings, midlegs with subequal tibiae and tarsi, nymphal lamellae of gills I–VII with expanded apexes.

Distribution: China (Zhejiang Province).

### 3.3. Regulaneuria Cingulata (Navás, 1933) Comb. Nov.

*Thalerosphyrus cingulatus* Navás, 1933: 18. Types: adult, from Chusan, Zhejiang, China.

*Thalerosphyrus cingulatus*: Wu, 1935: 252; Ulmer, 1935–1936: 215 [27]; Gui, 1985: 86; Zhou, 2013: 195; Zhou et al., 2015: 244; Sartori, 2014: 12.

*Compsoneuria cingulata*: Braasch and Soldán, 1986a: 46 (generic transfer); Webb, Braasch and McCafferty, 2006: 58.

Materials examined. Male imago (designed herein as neotype), Jiu-Feng Mountain (29°51′19.28″ N, 121°50′24.48″ E; alt. 62 m), Ningbo city, Zhejiang Province, 2021-VI-8-10, leg. Peng-Xu MU, De-Wen GONG; 15 male imagoes, 28 female imagoes and 10 larvae, same as neotype; 20 male imagoes, 4 male subimagoes, 15 female imagoes, 2 female subimagoes and 18 larvae, Jing-Tou village (27°30′44.11″ N, 120°29′37.51″ E; alt. 80 m), Wenzhou city, Zhejiang Province, 2021-VII-2-3, leg. De-Wen GONG.

Description:

Nymph: (in alcohol, Figure 1, Figure 2 and Figure 3 and Figure 8A): body length 6.0–8.0 mm, caudal filaments 7.0 mm, body yellowish-brown to brown (Figure 1A). Head capsule near sub-oval but posterior margin nearly straight, 2–3 pale dots located near base of antennae (Figure 1A,D).

Labrum ca. 0.5× width of head, with round apexes; anterior margin with shallow median groove, both surfaces with dense and long setae but those on dorsal surface much longer and denser (Figure 2A). Both mandibles covered with numerous setae on outer margins; prostheca with 8–12 fimbriate bristles (Figure 2D,E,H,I); outer incisor of left mandible with serrated margin and one larger terminal denticle; inner incisor subequal to outer one in length and with bifurcated apex (Figure 2D,H); outer incisor of right mandible serrated and with two apical terminal denticles; inner incisor divided into two sharp denticles too (Figure 2E,I). Hypopharynx: superlinguae curved and extended into blunt apex, row of hair-like setae on lateral margins from base to sub-apex; lingua bell-like, shorter than superlinguae in length and with tuft of short setae at apex (Figure 2B). Maxillae with scattered setae on ventral surface (Figure 2F,G), row of 13 comb-shaped setae on crown of galea-lacinia; two distal dentisetae arise from galea-lacinia independently; first one longer than others, second one divided into two branches; proximal dentiseta bifurcated, smaller one fringed; scattered setae on ventral surface of maxillae fimbriate (Figure 3A−C); first segment of maxillary palpi with sparse setae on outer margin; second segment obviously longer than basal one, outer margin with long setae, terminal segment with dense setaceous brush (Figure 2F). Labium: glossae slightly rhomboid, inner margin with tuft of long setae; paraglossae with dense hair-like setae and bristle-like setae on dorsal and anterior margins; labial palpi broad, ventral surface with setae brushes and scattered setae, similar setae on margins of labial palpi (Figure 2C).

Nota of thorax yellowish brown with pale markings, posterior tips of wingpads dark; sterna of thorax pale (sub-imaginal brown stripes visible in some mature nymphs) (Figure 1A,B); pronotum extended laterally, obviously wider than head (Figure 1A,D). Supracoxal spurs rounded (Figure 1E). Color pattern of all femora similar: three yellowish brown stripes washed at base, middle and apex, respectively, two additional dark brown dots at middle and apex; tibiae yellowish, tarsi of all legs slightly browner than tibiae; femora of all legs with long setae on outer margins, dorsal surfaces and inner margins with spatulate setae (Figure 4A–C). Foretibiae length 0.85× of femora, outer margin with short hair-like setae at base (Figure 4A), inner margin with row of bristle-like setae only; foretarsi length ca. 1/3 of tibiae, outer and inner margins with tiny hair-like setae. Midlegs similar to forelegs, except tibiae 0.79× length of femora (Figure 4B). Hindleg tibiae 0.82× of femora, outer margin with rows of dense hair-like setae and bristles, dorsal surface with row of bristle-like setae (Figure 4C,D). Claws of all legs with 3–4 subapical denticles (Figure 4E).

Abdominal terga I–X yellowish brown, with two median and two sub-lateral pale dots, but those dots on terga VIII–X fused together forming a larger one (Figure 1A). Sterna I–II with brown stripes on anterior margins (Figure 1B). Posterolateral angles of terga V–VII extended into small acute projections (Figure 1B,C). Lamellae of gills I broad with blunt apexes (Figure 4F); gills II–IV lamellae greatly expanded, posterior apexes rounded, with more fibrilliform than gills I (Figure 4G), lamellae of gills V–VI heart-like, with small blunt apexes (Figure 4H); gills VII near leaf-like in shape, with fine marginal setae while without fibrillae (Figure 4I). Caudal filaments pale to yellowish, with whorled spines on articulations (Figure 1A and Figure 8A).

Male imago: (in alcohol, Figure 5A, Figure 6, 7Figure A–E and Figure 8B): Body length 6.0–8.0 mm, forewings 7.0–8.0 mm, hindwings 1.5–2.0 mm, cerci 16.0–19.0 mm. Compound eyes clearly separated, distance between them 3× width of median ocellus (Figure 6A).

Mesonotum with apparent transverse suture, medial depression of furcasternum parallel (Figure 6B,C); pronota, mesonota and metanota with dark brown posterior margins, respectively, those stripes extended to coxa of all legs (Figure 6B,C); mesosternum and metasternum with dark brown posterior margins (Figure 6B). All legs with similar color pattern: femora reddish yellow to reddish brown, with three dark brown dots at base, middle and apex; tibiae yellowish brown with dark brown apex; tarsi yellowish (Figure 7A–C). Forelegs: length ratio of femora:tibiae:tarsi = 2.3:2.2:3.9, tarsal segments from basal to apical = 0.7:1.0:1.1:0.8:0.3 (Figure 7A). Midlegs: length ratio of femora:tibiae:tarsi = 1.9:1.4:1.7, tarsal segments arranged in decreasing order as 1, 2, 3, 5, 4 (Figure 7B). Hindlegs: length ratio of femora:tibiae:tarsi = 2.1:1.6:1.5, tarsal segments arranged in decreasing order as 1, 2, 3, 5, 4 (Figure 7C). All claws of legs similar, one blunt and one hooked.

Forewings hyaline but with dark tips, C, Sc and R1 fields with slightly thickened and brown crossveins; all crossveins washed with yellowish brown pigments, especially those of C and Sc fields; 13 crossveins in C section, those of Sc Sections 9–10, those of other regions arranged into five regular rows; Rs and MP forked at same level at wing base, MA forked over 1/2 distance from base of wing to margin (Figure 6D). Hindwings transparent but with remarkable brown stain on outer margins; costal margins slightly waved, blunt costal projection near base, MP forked in middle, MA forked much apically than MP (Figure 6E,F).

Abdominal terga I–IX yellowish, with dark brown stripes on posterior and lateral margins (Figure 6A). Sterna I–III with dark brown stripes (Figure 6B). Genitalia: styliger plate convex and with two distinct finger-like projections laterally (Figure 7D,E and Figure 8B). Forceps segment III subequal to segment IV in length (length ratio of segment III:segment IV = 1.1:1), and combination segments III–IV slightly shorter than half segment II. Penes almost totally fused totally but with apical incision, penial lobe round. Titillators present in subapical and medial position on penis lobes (Figure 7E and Figure 8B). Cerci yellowish, with brown dots on articulations.

Female imago: (in alcohol, Figure 5B, Figure 7F and Figure 8C,D): Body length 7.0–10.0 mm, forewings 8.0–10.0 mm, hindwings 2.0–3.0 mm, cerci 19.0–25.0 mm. Similar to male imago except as follows: length of femora:tibiae:tarsi of forelegs = 2.6:2.2:2.6, length order of tarsal segments arranged in decreasing order as 2, 1, 3, 4, 5; length of femora:tibiae:tarsi of midlegs = 2.6:2.0:1.7, tarsal segments arranged in decreasing order as 1, 2, 3, 5, 4; length of femora:tibiae:tarsi of hindlegs = 2.8:2.2:1.6, tarsal segments order similar to midlegs. Abdominal terga I–IX with reddish brown longitudinal stripes medially (Figure 5B). Subgenital plate reaching posterior margin of sternum VIII (Figure 7F and Figure 8C), posterior margin of subanal plate extended remarkably into a straight lobe, lateral margins of it thickened into ridges dorsally (Figure 7F and Figure 8C,D).

Male subimago: (in alcohol): body length 6.0–7.0 mm, forewings 6.0–7.5 mm, hindwings 1.5–2.0 mm, cerci 16.0–18.0 mm. Femora:tibiae:tarsi of forelegs = 1.8:1.2:2.0, tarsal segments arranged in decreasing order as 3, 4, 2, 1, 5; length of femora:tibiae:tarsi of midlegs = 1.9:1.2:1.3, length order of tarsal segments in decreasing order as 1, 2, 3, 5, 4; length of femora:tibiae:tarsi of hindlegs = 2.0:1.4:1.2, tarsal segments similar to midlegs. Color pattern similar to male except wings semi-hyaline and body paler.

Female subimago: (in alcohol): body length 10.0 mm, forewings 11.0 mm, hindwings 3.0 mm, cerci 19.0 mm. Body dull, color pattern similar to female imago. Femora:tibiae:tarsi of forelegs = 2.5:2.0:2.0, tarsal segments arranged in decreasing order as 2, 1, 3, 5, 4; length of femora:tibiae:tarsi of midlegs = 2.2:2.0:1.5, length order of tarsal segments in decreasing order as 1, 5, 2, 3, 4; length of femora:tibiae:tarsi of hindlegs = 2.3:2.0:1.4, tarsal segments similar to midlegs.

### 3.4. Ecology

**Figure 10:**
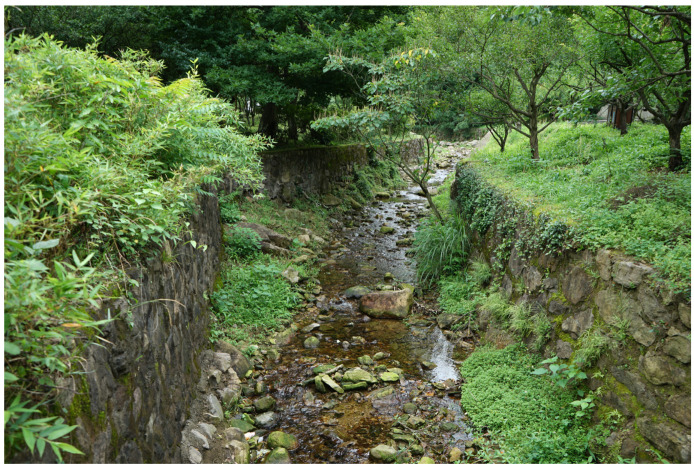
Collection locality of *Regulaneuria cingulata*.

Nymphs of this species live in small creeks with water 0.3−1.2 m wide and 0.1−0.3 m deep (Figure 10). Most of the collected nymphs are found on the surface of stones in moderate to fast flowing water. The observed last instar female nymphs molt at 14:00–15:00 p.m. local time. The nuptial flight was observed at 12:00–15:30 p.m.

### 3.5. Affinity

Although the characteristics of *R. cingulata* deserves the establishment of a new genus, its phylogenetic position is unclear. First, the reduced crossveins, fused penes and extended female subanal plate are clear apomorphies, but those characters can be found in several different genera of the same subfamily (e.g., crossveins reduction in *Compsoneuria*, fused penes in *Asionurus* and *Thalerosphyrus*, long subanal plate in *Asionurus*). Second, some other morphological characters, such as longer tarsi, colorful wings and widely separated compound eyes (maybe originated from smaller eyes), are obvious plesiomorphies and they also distribute in several other genera of Heptageniidae (for instance, long tarsi in *Thalerosphyrus*, small eyes in *Stenacron* and pigmented wings in *Atopopus*). Third, the nymphal structures, such as mouthparts and gills, are very variable and hard to use in phylogeny reconstruction at generic level of the Heptageniidae. Considering its nymphal morphology, this new genus is close to *Asionurus* but its imaginal morphology is similar to the tribe Compsoneuriini. The eggs of *Regulaneuria*, which have moderate number and size of KCTs, show some uniqueness too although we can say certainly that it is a member of the subfamily Ecdyonurinae.

## 4. Discussion

In the original description of Navás on *Thalerosphyrus cingulatus* males, the tarsal segment I is longer than the second one of foreleg, and the tibia is slightly longer than femur in hindlegs [1]. These two points are not true compared to our fresh materials. Our specimens show that the first tarsal segment is ca. 0.7× length of the second, hind tibia is about 0.8× length of femur. Further, nor is the original drawing on forewing venation of Navás exactly showing the fact. In our view, those differences maybe result from the low quality of microscope or inaccurate observation at that time.

Braasch and Soldán showed that the crossveins on forewings of an Indonesian mayfly *Compsoneuria diehli* Braasch and Soldán, 1986b are all pigmented [22]. However, its penes are separated widely, its crossveins are not aligned into any regular rows and hindtarsus is much shorter than tibia. Further, its nymphs are not described. So here we follow the judgement of Sartori that it needs more research to confirm its real status [8].

In the original description of Eaton (1883–1888) [28], the forewing of *Compsoneuria spectabilis* Eaton, 1881 has few rows of crossveins, which is very similar to *R*. *cingulata*. However, in the re-description on the former species provided by Sartori [8], it has more crossveins actually. On the other hand, Braasch and Boonsoong reported that the species *Compsoneuria langensis* Braasch and Boonsoong, 2010 and *C. perakensis* Braasch and Boonsoong, 2010 have colorful wings and less crossveins [29], but they never line up to regular rows. Similarly, Braasch and Soldán showed that the crossveins on forewings of *Asionurus petersi* Braasch and Soldán, 1986b are all pigmented but they are still random distributed [22]. The forewings of species *Compsoneuriella thienemanni* Ulmer, 1939 also were shown some pigmented crossveins, but they are irregularly distributed [8,11]. In short, in Heptageniidae, reduction in the number of crossveins are found in several species but they are generally more numerous than in our new genus and irregularly situated.

Remarkably, some species in Leptophlebiidae and Baetidae show very alike crossveins and color pattern to *R*. *cingulata*. For example, leptophlebiid species *Atalomicria sexfasciata* Ulmer, 1916 and *A. bifasciata* Campbell and Peters from Australia have heavily pigmented and reduced crossveins on their forewings [30,31]. The latter species even have the similar crossvein rows to *R. cingulata*. An Asian species *Baetiella bispinosa* (Gose, 1980) of Baetidae also has less and pigmented crossveins [32,33]. Obviously, those similarities are homoplasies and results of convergent evolution.

Another point to mention is that although the crossveins reduction can happen in different lineages of Heptageniidae, the number of crossveins of them is still much more than some other families, similar to most Baetidae (such as *Baetiella* Ueno, 1931, *Baetis* Leach, 1815 and *Nigrobaetis* Novikova and Kluge, 1987) [33,34,35], Caenidae (e.g., *Caenis* Stephens, 1835, *Brachycercus* Curtis, 1834 and *Sparbarus* Sun and McCafferty, 2008) [36,37,38], Prosopistomatidae (for instance, *Prosopistoma* Latreille, 1833) and Oligoneuriidae (such as *Oligoneuriella* Ulmer, 1924) [39,40], which almost lost all crossveins. This maybe originate from their relatively bigger body and wings (*R. cingulata* around 8.0–10.0 mm). As a general rule, the mayflies having shorter or tiny body (less than 5.0 mm) usually have less crossveins. This is very clear in Baetidae and Caenidae.

The position and numbers of left or remained crossveins in forewings (excluding those in C and Sc sections) of our new genus are consistent with those of similar baetid and leptophlebiid counterparts. This maybe result from they play key roles in connecting longitudinal veins and in flight, which prevent them from further reduction or loss unless they reduced their bodies further.

In our observation, the mayflies that are more active in daytime usually have more colorful wings and bodies [41]. In our observation, the adults of *R. cingulata* perform mating flight around noon and afternoon (12:00–15:30). At the same time, their wings and bodies are very conspicuous and colorful. However, we do not know whether this phenomenon is a general rule or just random situations.

## Data Availability

All data is available in this paper.
